# Primary external iliac vein leiomyosarcoma in a young female diagnosed by contrast-enhanced ultrasound: A case report

**DOI:** 10.1097/MD.0000000000022512

**Published:** 2020-12-11

**Authors:** XiaoChen Shi, JunXue Gao, WanLei Zhang

**Affiliations:** Department of Ultrasound, Peking University People's Hospital, Beijing, China.

**Keywords:** contrast-enhanced ultrasound, deep vein thrombus, prognosis, venous leiomyosarcoma

## Abstract

**Rationale:**

Venous leiomyosarcoma (LMS) is a malignant tumor arising from the smooth muscle cell of the vein wall. The diagnosis of venous LMS is usually delayed owing to its rarity, absence of serological markers, and mimicry with deep vein thrombosis (DVT). Herein, we report a case of a primary external ilian vein LMS characterized by long-term, unexplained DVT in the left lower limb. Contrast-enhanced ultrasound (CEUS) played a crucial role in the preoperative diagnosis. No improvement was observed in the lower limb status; a rapid, high-level, heterogeneous wash-in and wash-out mass of the vein, as seen with CEUS, could indicate angiogenic malignancy. CEUS also helped evaluate the percent of intratumoral necrosis, which is an important parameter for predicting the prognosis.

**Patient Concerns:**

A 37-year-old Chinese women presented to the Vascular Surgery Department of our hospital for accurate diagnosis of her condition. She began experiencing edema and pain in her left leg 2 years ago. She was diagnosed with DVT in the left lower extremity and was administered anticoagulant therapy since then. However, her symptoms started to aggravate 8 months ago.

**Diagnoses:**

The laboratory results including D-dimer, prothrombin time (PT), activated partial thrombin time (APTT), and prothrombotic conditions screening were within normal ranges. A pelvic ultrasound detected a heterogeneous, hypoechoic mass compressing the external iliac vein and obstructing the venous drain of the lower extremity. The mass showed a rapid, high-level, heterogeneous wash-in and wash-out on CEUS, which suggested angiogenic malignancy. Contrast-enhanced CT (CECT) confirmed the result of CEUS but revealed no metastasis.

**Interventions:**

She underwent complete surgical removal of the tumor, which was resected successfully. There was no infiltration in the inguinal nodes sent for the study.

**Outcomes:**

Pathological examination and immunohistochemistry confirmed that the mass was a well-differentiated LMS originating from the external iliac vein. There was no sign of local recurrence or distant metastasis during a 12–month follow-up.

**Lessons:**

Effective imaging techniques and differential diagnosis of venous LMS is vital and should be considered for patients with chronic thrombosis presenting with normal laboratory results.

## 1 Introduction

Primary tumors of the peripheral veins are rare lesions, and they are generally malignant. LMS is the most common pathologic variant. Venous LMS is an aggressive tumor with a high rate of early hematogenous metastasis and local recurrence.^[1,2]^ To date, 300 cases of venous LMS have been reported previously, which involves the inferior vena cava (IVC) in approximately 2 third of the cases. Reports of LMS originating from the external iliac vein are especially rare, and to our knowledge only 3 cases have been reported.^[3,4,5]^ Venous LMS has clinical signs that may be confused with those of DVT, thus causing delay in the correct diagnosis and timely intervention. However, an unsuccessful clinical course of a presumed DVT should raise suspicion for an alternative diagnosis. Although gray-scale ultrasound and color Doppler flow imaging (CDFI) are widely used for characterizing the morphology and vascularization of solid masses, they lack specificity in diagnosing angiogenic tumors.^[6,7,8]^ We report a case of an external iliac vein LMS in a 37-year-old women diagnosed by CEUS. In our opinion, surgery should not be delayed after a diagnostic suspicion with the imaging test. We hope our report can facilitate the preoperative diagnosis of venous LMS.

## 2 Case report

Written informed consent for the publication of this case report was obtained from the patient. Ethics approval for this study was waived by the Ethics Committee of Peking University People's Hospital (Beijing, China) because it involved fewer than 3 patients.

A 37-year-old Chinese women (gravida 1 and para 1) who had suffered from edema and pain in the left extremity for 2 years was referred to the Vascular Surgery Department of our hospital for accurate diagnosis. She underwent an ultrasound examination at the local hospital 2 years ago and it suggested thrombi in her left femoral vein and superficial femoral vein. She had been administered anticoagulant therapy since the diagnosis. However, her symptoms started to aggravate 8 months ago. Therefore, she came to our hospital for further treatment. The laboratory results including D-dimer, prothrombin time (PT), activated partial thrombin time (APTT) and prothrombotic conditions screening were within normal ranges.

**Figure 1:**
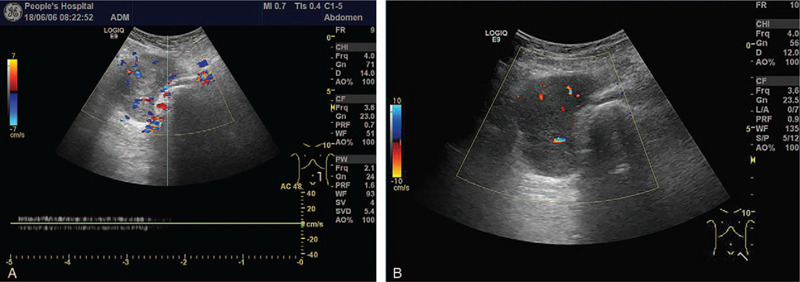
A A 6.4 ×  5.0 × 4.7 cm heterogeneous, hypoechoic mass was seen around the left external iliac vein. The shape of the mass was regular and the boundary of the mass was clear. The relationship between the mass and left external iliac vein was blurred. 1B CDFI detected a few color spots in the mass.

**Figure 2:**
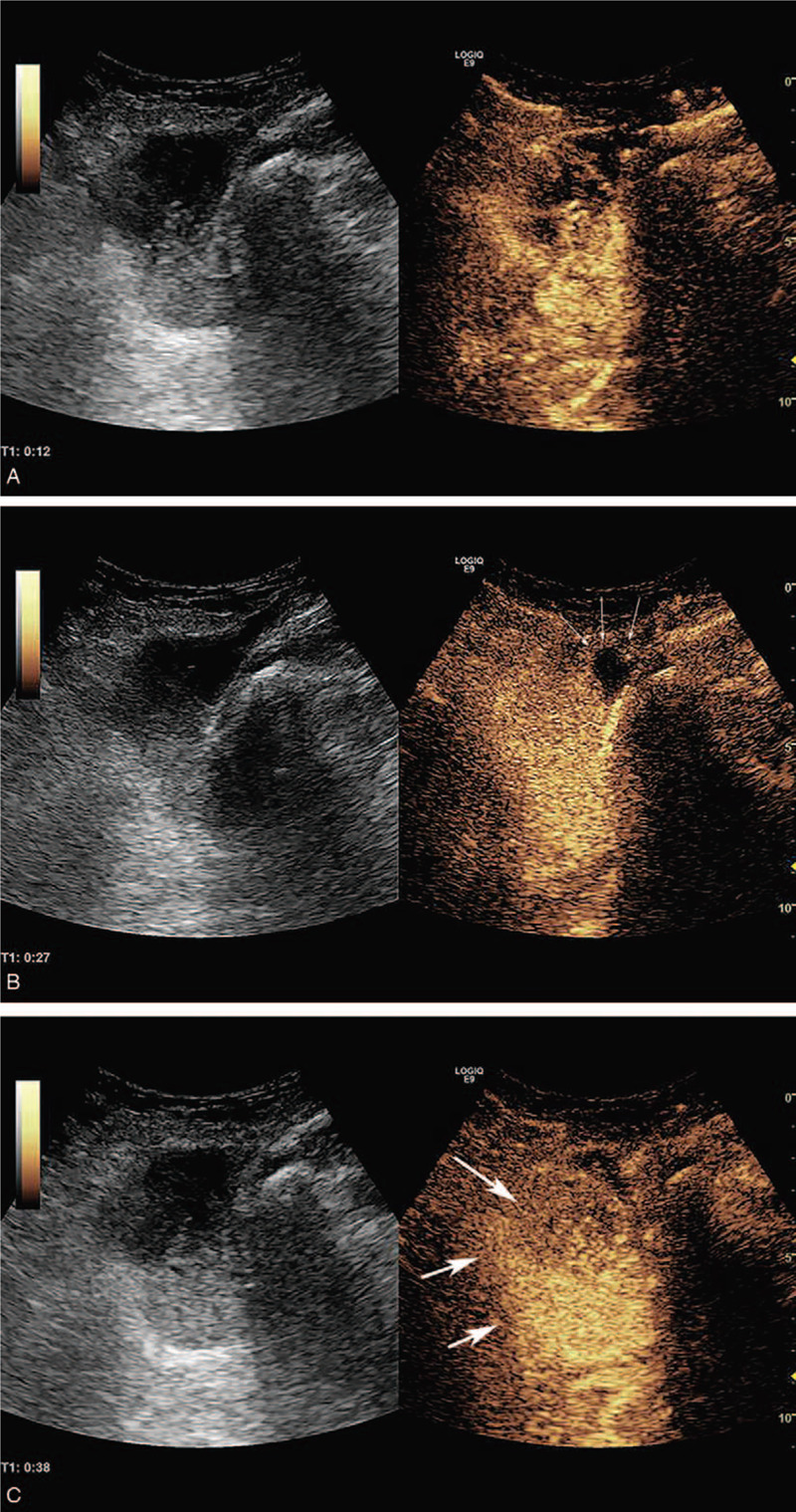
A The mass began to enhance at 12 s with a rapid, heterogeneous wash-in. 2B The mass peaked at 27 with a high-level, heterogeneous enhancement, an unenhanced area about 1 cm in diameter was seen in the anterior portion of the mass (arrows). 2C the mass began to subside with a low-level, heterogeneous enhancement at 38 s, a clear margin was seen during the examination (arrows).

**Figure 3:**
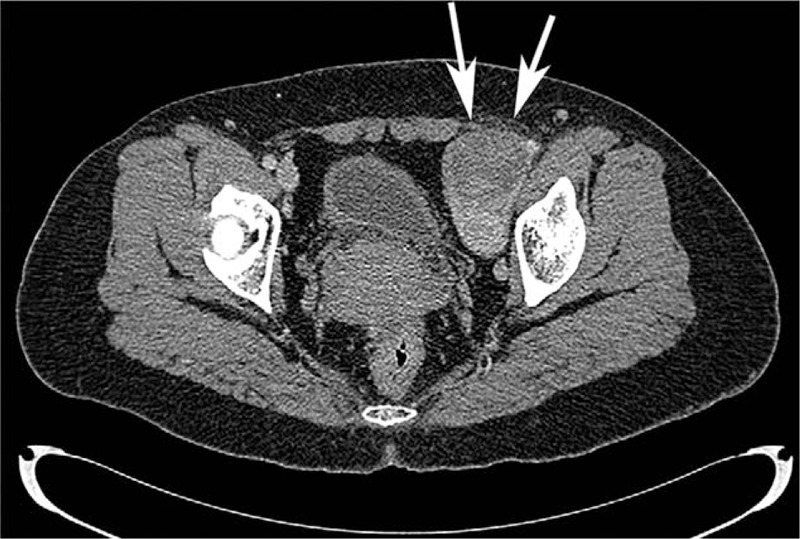
Axial contrast-enhanced venous phase CT showed an enlargement of her left external iliac vein due to a heterogeneous enhanced mass measuring7.5 × 6.0 × 5.7 cm (arrows).

Vascular ultrasound at our hospital suggested thrombi in the superficial femoral and femoral vein of her left extremity. It also suggested venous stasis in the deep vein in the calf of her left leg. Based on the ultrasound performance and laboratory results, the possibility of compression of her left external iliac vein was considered. Her external iliac vein was examined; the distal section of the external iliac vein was not continual. A 6.4 × 5.0 × 4.7 cm heterogeneous, hypoechoic mass was seen around the left external iliac vein. The shape of the mass was regular, and the boundary of the mass was clear. The relationship between the mass and left external iliac vein was blurred (Fig. 1A). CDFI detected a few color spots in the mass (Fig. 1B). Accordingly, CEUS (GE Logiq E9, GE Healthcare, USA) was performed for further characterization after an injection of 4 ml of Sonove (Bracco, Milan, Italy). The mass began to enhance at 12 s with a rapid, heterogeneous wash-in (Fig. 2A). The mass peaked at 27 s with a high-level, heterogeneous enhancement, and an unenhanced area measuring about 1 cm in diameter was seen in the anterior portion of the mass (arrows)(Fig. 2B). The mass began to subside with a low-level, heterogeneous enhancement at 38 s; a clear margin was seen during the examination (arrows) (Fig. 2C). Combined gray-scale ultrasound, CDFI, and CEUS findings were suggestive of a malignant tumor originating from the left external iliac vein. The patient underwent CECT for further confirmation. Axial contrast-enhanced venous phase CT showed a dilation of her left external iliac vein due to a heterogeneous enhanced mass measuring 7.5 × 6.0 × 5.7 cm, and the enhanced CT value was 43 Hu-101 Hu (Fig. 3); metastases were not detected. Based on the CECT findings, diagnosis of LMS arising from the external iliac vein was concluded.

**Figure 4:**
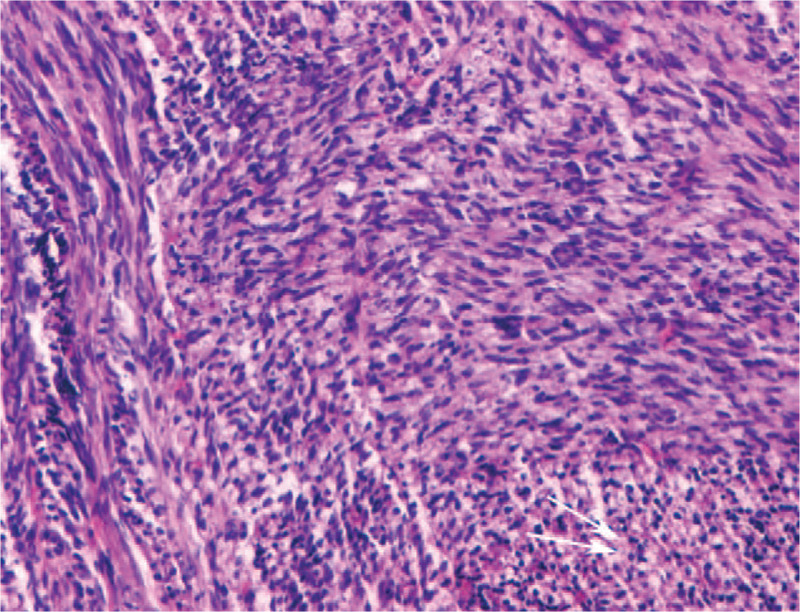
Pathological examination revealed spindle-shaped cells arranged in fascicles. Mitotic activity was 3–5 mitoses/10 HPF (high power field) with occasional atypical mitotic figures (arrows).

The patient underwent surgery, during which a solid, flesh-like mass with a clear pseudocapsule was found in her left iliac vein, causing an obvious enlargement of the lumen. Grossly, the mass measured 8.0 × 7.5 × 6.5 cm. Pathological examination revealed spindle-shaped cells arranged in fascicles. Mitotic activity was 3–5 mitoses/10 HPF (high power field) with occasional atypical mitotic figures (Fig. 4). Immunohistochemistry results were as follows: SMA (+), EMA (focal+), Desmin (focal+), Vimentin (+), Calesmon (slight+), CK (-), ER (-), Ki-67 (10%+), S-100 (-), CD34 (+), CD31 (+). A well-differentiated leiomyosarcoma originating from the external iliac vein was identified. Infiltration of the inguinal nodes was not detected in the specimen sent for diagnosis. The patient underwent postoperative radiotherapy for 6 weeks. During an 18-month follow-up, there was no sign of local recurrence or distal metastasis.

## 3 Discussion

Primary venous LMS is an aggressive, malignant tumor arising from the smooth muscle cell of the vein wall.^[9]^ Venous LMS accounts for 5% of soft tissue LMS.^[10]^ Abed et al^[11]^ and Berlin et al^[12]^ concluded that majority of the extremity venous LMSs originate from lower extremities. Unlike LMSs of IVC, which have a preponderance with 6:1 ratio of females vs males,^[13]^ LMSs of peripheral veins do not show such preference as reported in the literature; these tumors occur in adults older than 55 years old.^[2,14]^ In the present case, venous LMS occurred in a 37-year-old women, which is the youngest patient in the reported literature.

The clinical manifestation of a venous LMS is nonspecific. It primarily depends on the growth pattern, size, site, growth rate of the tumor, and relationship with the surrounding tissues.^[10]^ The growth pattern of venous LMS vary from intraluminal (5%) to extraluminal (62%) to mixed forms (33%). Intraluminal venous LMS presents early with vague symptoms of impaired venous circulation such as thrombus, edema, pain or Budd-Chiari syndrome. However, extraluminal venous LMS accounts for the majority of these tumors, and such symptoms do not occur until the tumor grows to a large size. The laboratory results and serological markers were within normal ranges, and thus, the diagnosis is usually neglected. According to Abed et al,^[11]^ most patients were misdiagnosed initially because of its poorly distinguished clinical symptoms and its similarity with DVT.

Generally, LMSs are hypervascular and have a pseudocapsule. LMSs are prone to develop central hemorrhage, necrosis, and cystic areas.^[14]^ The etiology remains unclear, although it has been reported that LMS is highly probable in immunosuppressed patients (HIV-positive patients and organ transplant recipients).^[15]^ In this case, the patient tested negative for HIV serology, and there was no significant medical evidence that indicated the possibility of immunosuppression. The histological variant of sarcoma does not provide sufficient information for planning therapy and predicting prognosis. According to the French Federation of Cancer Centers Sarcoma Group criteria (Table 1),^[16]^ histologic grade is the best indicator for overall survival and prognosis prediction, which is based on tumor differentiation, mitotic rate, and percent of tumor necrosis.

**Table 1:** Federation nationale des centres de lutte contre le cancer grading system criteria.

Characteristic	Finding
Tumor differentiation	
Score 1	Sarcomas closely resembling normal adult mesenchymal tissue (e.g., well-differentiated liposarcoma)
Score 2	Sarcomas for which histologic typing is certain (e.g., myxoid liposarcoma) Embryonal and undifferentiated sarcomas, sarcomas of doubtful type, synovial sarcomas, osteosarcomas, primitive neuroectodermal tumor

Mitotic count^∗^	
Score 1	0–9 mitoses per 10 high-power fields
Score 2	10–19 mitoses per 10 high-power fields
Score 3	20 mitoses per 10 high-power fields
Tumor necrosis	
Score 0	No necrosis
Score 1	<50% tumor necrosis
Score 2	>50% tumor necrosis
Histologci grade	
Grade 1	Total score 2, 3
Grade 2	Total score 4, 5
Grade 3	Total score 6, 7, 8

∗ High-power field measures 0.1734 mm^2^.

In our case, the patient was diagnosed as having left lower limb DVT 2 years ago and received administered anticoagulant therapy since then. However, her symptoms started to worsen 8 months ago. The laboratory results were within normal ranges. The above findings led us to consider the possibility of compression of iliac vein, obstructing the venous drain of the lower extremity. Unlike the natural history of DVT in lower limbs, when a venous LMS is involved, venous obstruction symptoms are not relieved after anticoagulant therapy; DVT symptoms are prone to worsen due to the increase in tumor size. A pelvis ultrasound detected a heterogeneous, hypoechoic mass compressing the left iliac external vein. These findings validated our assumption. Our case highlights the possibility of angiogenic tumor in the differential diagnosis of a swelling leg, especially when the laboratory results are within normal ranges. We also consider that ultrasound functions as the first choice for vascular examination; the iliac vein should be routinely examined for patients with DVT of lower extremity.

Ultrasonography is useful for the detection of the morphology and vascularization of solid masses. In our case, ultrasonography revealed a hypoechoic, heterogeneous mass and CDFI detected a few color spots within the mass, which is consistent with the previously reported literature.^[6,8]^ However, CDFI is not sensitive enough for the detection of intratumoral microvessels or tissue perfusion;^[17,18]^ further examination needs to be performed for detailed information. CEUS is regarded as a supplementary modality to CDFI and allows real time imaging for the evaluation of tissue perfusion.^[19]^ More importantly, studies have demonstrated that deep vein systems can be visualized more clearly with ultrasound contrasts.^[20]^ To date, there is only one report describing CEUS imaging features for the diagnosis of venous LMS.^[21]^ The CEUS imaging characteristics in our case and literature both demonstrate a rapid, high-level, heterogenous wash-in and wash-out, which is probably caused by tumor heterogenous perfusion or necrosis. In this case, there was a clear margin during the enhancement, and this may correspond to the pseudocapsule of the tumor.

Venous LMS is rare. Given the rarity of literature on the tumor and lack of robust data on the imaging diagnosis, venous LMS can be easily misdiagnosed as a benign angiogenic tumor, such as leiomyomatosis. B-mode ultrasound lack specificity in the differential diagnosis between LMS and leiomyomatosis since they can both present as a hypoechoic mass. According to Gaetke et al,^[6]^ heterogeneity is a typical feature for the differential diagnosis between leiomyomatosis and LMS. In our case, CEUS turned out to be helpful in revealing the intratumoral heterogeneity. According to Hollenbeck et al,^[14]^ LMSs of the IVC are usually larger than 10 cm, and they are prone to develop large amount of central necrosis, which corresponds to a non-enhanced area on CEUS. However, only a small non-enhanced area measuring 1 cm in diameter in the anterior portion was found in the mass; this is probably because the tumor was well-differentiated and the growth in the lumen was slow. According to the French Federation of Cancer Centers Sarcoma Group criteria (Table 1),^[16]^ percent of tumor necrosis is a parameter to predict prognosis. In our case, the percent of non-enhanced area was less than 50%, which was score 1. The well-differentiation of the tumor was score 1; the mitotic count of the tumor was score 1; the total score of the tumor was score 3 and grade 1, which indicated a better prognosis.

Both CEUS and CECT findings revealed the intratumoral heterogeneous perfusion. Our experiences suggest that CEUS is compatible with CECT in distinguishing benign and malignant tumors in a vein. Compared with CECT, CEUS is superior in revealing hemodynamic information and showing local details of peripheral blood vessels.^[22]^ However, since CEUS is based on B-mode ultrasound, it has limitations, such as obesity, intestinal gas, and deep site origin. In our case, CEUS failed to reveal the enlargement of external iliac vein due to the large size and deep origin site of the tumor. CECT was used to confirm the result of CEUS. CECT also completed the data regarding extension of the tumor, its relationship with surrounding tissues, and metastasis.

In our opinion, biopsy should be avoided to reduce the possibility of dissemination. Since contrast-enhanced imaging modalities can provide sufficient information of the tumor, maximal surgical resection should no longer be delayed.^[23]^ Venous LMS is considered as an aggressive tumor with a high rate of early hematogenous metastasis, since there is direct access to the blood stream; local recurrence is also involved.^[1,2]^ Postoperative radiotherapy and chemotherapy may be helpful in controlling the local recurrence in case of incomplete tumor resection. Our patient underwent radical surgery followed by postoperative radiotherapy and has survived 18 months without any local recurrence or metastasis.

## 4 Conclusion

The clinical signs of venous LMS are nonspecific and misleading. Heightened clinical awareness and effective imaging enable the correct diagnosis and timely intervention. In patients with unexplained lower limb DVT, contrast-enhanced imaging modalities should be used for confirming the pathology and rule out metastasis. When a sarcoma is diagnosed, it is advisable to re–evaluate the percent of necrosis with CEUS for the prediction of prognosis. Owing to the rarity of this condition, clinicians and radiologists have limited experience. We hope our report provides data to the literature for the better diagnosis of this entity.

## Acknowledgment

The authors thank Editage (www.editage.com) for English language editing.

## Author contributions

XiaoChen Shi has collected the information and wrote the manuscript.

JunXue Gao has performed the ultrasound examination.

WanLei Zhang has dealt with the language.

## References

[1] Roland CL Boland GM Demicco EG Primary vascular leiomyosarcoma: clinical observations and molecular variables. JAMA Surg 2016;151:347–54. 26629783 10.1001/jamasurg.2015.4205 PMC4941943

[2] Reix T Sevestre H Sevestri-Pietri MA Primary malignant tumors of the venous system in the lower extremities. Ann Vasc Surg 1998;12:586–96. 10.1007/s100169900205 9841691

[3] Sahu R Aggarwal R Kumar P Primary external iliac vein leiomyosarcoma. Ann Vasc Surg 2019;57:274.e5–e9. 10.1016/j.avsg.2018.09.030 30500633

[4] Fukuda W Taniguchi S Fukuda I Leiomyosarcoma of the external iliac vein. Vasc 2012;20:178–80. 10.1258/vasc.2011.cr0305 22499616

[5] Tripodi E Zanfagnin V Fava C Leiomyosarcoma of the right iliac veins presenting as a pelvic mass: a case report. Obstet Gynecol Cases Rev 2015;2:40–4.

[6] Gaetke-Udager K McLean K Sciallis AP Diagnostic Accuracy of Ultrasound, Contrast-enhanced CT, and Conventional MRI for differentiating leiomyoma from leiomyosarcoma. Acad Radiol 2016;23:1290–7. 27396800 10.1016/j.acra.2016.06.004

[7] Hemant D Krantikumar R Amita J Primary leiomyosarcoma of the inferior vena cana1: imaging features. J Clin imag 2015;2:274–8. 10.1046/j.1440-1673.2001.00955.x 11903177

[8] Bousquet JC Goze A Hassan M Leiomyosarcoma of the inferior vena cava. Ultrasonographic appearance. J Ultrasound Med 1987;6:7–12. 3546727 10.7863/jum.1987.6.1.7

[9] Cho SW Marsh JW Geller DA Surgical management of leiomyosarcoma of the inferior vena cava. J Gastrointest Surg 2008;12:2141–8. 18841423 10.1007/s11605-008-0700-y

[10] Tilkorn DJ Hauser J Ring A Leiomyosarcoma of intravascular origin: a rare tumor entity:clinical pathological study of twelve cases. World J Surg Oncol 2010;8:103. 21092216 10.1186/1477-7819-8-103 PMC3012034

[11] Abed R Abudu A Grimer RJ Leiomyocarcomas of vascular origin in the ectremity. Sarcoma 2009;2009:385164. 19587823 10.1155/2009/385164 PMC2705766

[12] Berlin O Stener B Kindblom LG Leiomyosarcomas of venous origin in the extremities. A correlated clinical, roentgenologic, and morphologic study with diagnostic and surgical implications. Cancer 1984;54:2147–59. 6488138 10.1002/1097-0142(19841115)54:10<2147::aid-cncr2820541015>3.0.co;2-9

[13] Dzsinich C Gloviczki P Van Heerden JA Primary venous leiomyosarcoma: a rare but lethal disease. J Vasc Surg 1992;17:595–603. 1560548

[14] Hollenbeck ST Grobmyer SR Kent KC Surgical treatment and outcomes of patients with primary inferior vena cava leiomyosarcoma. J Am Coll Surg 2003;197:575–9. [13] Juan R.Ackerman's surgical pathology. Elsevier, 2014. 14522326 10.1016/S1072-7515(03)00433-2

[15] Xu J Velayati A Berger BJ Leiomyosarcoma of the inferior vena cava in an HIV-Positive adult patient: a case report and review of the literature. Am J Case Rep 2017;18:1160–5. 29097650 10.12659/AJCR.905787 PMC5683681

[16] Fletcher CDM Unni KK Mertens F Pathology and Genetics of Tumors of Soft Tissue and Bone. World Health Organization Classification of Tumors; Vol 5. Lyon, France: IARC; 2002.

[17] Enzinger FM Weiss S W “Leiomyosarcoma”. Soft Tissue Tumors Eds 2014;6:561–5.

[18] Székely E Kulka J Miklós I Leiomyosarcomas of great vessels. J Pathol Oncol Res 2000;6:233–6. 10.1007/BF03032379 11033466

[19] Greis C Technology overview; SonoVue (Bracco, Milan). Eur Radiol 2004;14: suppl 8: 11–5. 15700328

[20] Lassau N Koscielny S Opolon P Evaluation of contrast-enhanced color Doppler ultrasound for the quantification of angiogenesis in vivo. Invest Radiol 36:50–5. 11176261 10.1097/00004424-200101000-00007

[21] Zhang M Feng Y Huang B Multimodal ultrasonographic assessment of leiomyosarcoma of the femoral vein in a patient misdiagnosed as having deep vein thrombosis. Medicine 2017;96:46–50. 10.1097/MD.0000000000008581 PMC5704814 29145269

[22] Sidhu PS Choi BI Nielsen MB The EFSUMB guidelines on the nonhepatic clinical applications of contrast-enhanced ultrasound (CEUS): a new dawn for the escalating use of this ubiquitous technique. Ultraschall Med 2012;33:5–7. 22322478 10.1055/s-0031-1299141

[23] Jose Gil-Sales Sandra Vicente Nuria Martinez Leiomyosarcoma of the deep femoral vein. A rare cause of venous obstruction in lower limbs and an alternative diagnosis to chronic venous thrombus. Ann Vasc Surg 2012;26:1013–6. 10.1016/j.avsg.2012.02.015 22944578

